# Manipulation of the Duration of the Initial Self-Control Task Within the Sequential-Task Paradigm: Effect on Exercise Performance

**DOI:** 10.3389/fnins.2020.571312

**Published:** 2020-10-08

**Authors:** Ruth Boat, Raymon Hunte, Emily Welsh, Anna Dunn, Ellen Treadwell, Simon B. Cooper

**Affiliations:** Sport, Health, and Performance Enhancement Research Centre, Department of Sport Science, Nottingham Trent University, Nottingham, United Kingdom

**Keywords:** ego depletion, pain, motivation, Stroop task, physical performance

## Abstract

Self-control exertion on an initial task has been associated with impaired performance on subsequent physical tasks also requiring self-control; an effect suggested to be mediated by changes in perceptions of pain and motivation. However, the effects of spending longer on the initial self-control task are unknown. This study, therefore, explored the potential for the duration of the initial self-control task to influence subsequent physical performance, perceptions of pain, and perceived motivation; particularly during the early stages of the physical task. In a within-subject design, 29 participants (11 male, 18 female) completed a wall-sit task until volitional exhaustion, on four separate occasions. Prior to each wall-sit, participants completed either a non-self-control task (congruent Stroop task) for 4 min, or a self-control task (incongruent Stroop task) for 4 (short duration), 8 (medium duration), or 16 (long duration) min. Participant’s perceptions of pain and motivation were recorded every 30 s during the wall-sit. Wall-sit performance time was analyzed using one-way ANOVA and perceptions of pain and motivation analyzed using multi-level modeling. Wall-sit performance time was significantly longer on the non-self-control exertion trial compared to all other trials (all *p* < 0.01), as well as longer on both the short duration and medium duration self-control exertion trials compared to the long duration self-control exertion trial (both *p* < 0.001). Perceptions of initial (at 30 s) pain and motivation were different between the trials (main effect of trial: pain, *p* = 0.001; motivation, *p* < 0.001); whereby longer durations of self-control exertion increased perceptions of pain and decreased motivation. The decrease in motivation during the wall-sit task was greater on the long duration self-control exertion trial compared to all other trials (trial^∗^time interactions, all *p* < 0.05). The present study provides novel evidence that spending longer on the initial self-control task led to greater detrimental effects on subsequent wall-sit performance time. Furthermore, longer duration self-control exertion tasks led to increased perceptions of pain and decreased motivation within the first 30 s of the wall-sit task, as well as a greater decrease in motivation across the wall-sit task. These attentional and motivational shifts may explain performance decrements following the exertion of self-control.

## Introduction

Self-control is defined as the ability to volitionally regulate dominant impulses or urges to bring them in line with more desirable, long-term goals (Baumeister et al., 1998). Self-control helps individuals to exhibit appropriate behavior by helping to regulate urges, juggle competing goals, and to maintain focus on the desired goal (Baumeister et al., 2007). High levels of self-control have been linked with numerous adaptive behaviors from a variety of contexts; including enhanced psychological well-being, higher levels of achievement and performance, and improved interpersonal relationships (e.g., Tangney et al., 2004; Baumeister et al., 2007; De Ridder et al., 2012). In addition, self-control has been shown to affect athletic performance (Englert, 2016), whereby it is essential for athletes to control their cognitive, emotional, and motor processes, in addition to their behavioral tendencies (Englert and Bertrams, 2012; Wagstaff, 2014).

The capacity to exert self-control can differ both between individuals (i.e., trait self-control), as well as across situations within the same individual (i.e., state self-control; Tangney et al., 2004). Concerning state self-control, recent meta-analytic evidence has emphasized that the initial exertion of self-control on one task, impairs performance on a subsequent, ostensibly unrelated task also requiring self-control (Hagger et al., 2010; Dang, 2017; Giboin and Wolff, 2019; Brown et al., 2020). However, a Registered Replication Report did not find support for this depletion effect (Hagger and Chatzisarantis, 2016); with some researchers suggesting that publication bias may have led to an overestimation of the size of the effect (Carter et al., 2015; Wolff et al., 2018). However, many recent commentaries, analyses, and debates have implied that although the size of the depletion effect is likely smaller than previously suggested, it is too early to reject the effect altogether (e.g., Baumeister and Vohs, 2016; Sripada et al., 2016; Blázquez et al., 2017).

Within the literature to date, the completion of various self-control tasks (e.g., completing an incongruent Stroop task, transcribing a neutral text while omitting the letters “e” and “n,” suppressing emotions during an upsetting movie) have impaired performance on subsequent physical tasks including a wall-sit task (Boat et al., 2018), cycling performance (Wagstaff, 2014; Englert and Wolff, 2015; Boat et al., 2017), press-up and sit-up tasks (Dorris et al., 2012), as well as skill-based tasks (Englert and Bertrams, 2012; McEwan et al., 2013). While it is important to note that there is some contrasting research (Hagger and Chatzisarantis, 2016), overall the evidence base suggests that self-control exertion seems to have a negative effect on subsequent physical performance (Giboin and Wolff, 2019; Brown et al., 2020).

The shifting priorities model (Inzlicht et al., 2014; Inzlicht and Schmeichel, 2016) has recently been applied to explain self-control failures in a multitude of performance contexts, including sport and exercise settings. The core assumption of this model is that following the exertion of self-control, individuals experience shifts in motivation and attention that undermines performance on subsequent tasks that also require self-control (Inzlicht et al., 2014; Inzlicht and Schmeichel, 2016). A number of physical tasks that have been employed in previous self-control research are unpleasant and induce elevated levels of discomfort and pain (e.g., Dorris et al., 2012; Englert and Wolff, 2015). An essential function of pain is to disturb and stimulate attention (Eccleston and Crombez, 1999). Thus, perceptions of pain during physically effortful tasks can be utilized as a measure of attentional shifts within the shifting priorities perspective (Boat and Taylor, 2017). For instance, following prior self-control exertion, recreationally active participants described higher perceptions of pain and decreased motivation during the initial stages of a wall-sit task, which resulted in reduced performance on the wall-sit task; relative to when they did not initially exert self-control (Boat and Taylor, 2017; Boat et al., 2018). Although initial evidence appears to support the shifting priorities model, further research is required to test the mechanisms of this model (Englert, 2019). For instance, examining changes in perceptions of pain and motivation to perform subsequent task goals, throughout a physical performance task, have not been examined to date, and would provide a novel insight into the mechanisms underpinning the shifting priorities model and how this affects subsequent performance.

Recent literature relating to the shifting priorities model of self-control is consistent with reward-based models of self-control, whereby individuals weigh the benefits of pursuing a specific task against its costs (Kurzban et al., 2013; Wolff and Martarelli, 2020). In other words, during an endurance task, individuals repeatedly appraise the pros and cons of decreasing or sustaining effort to perform optimally. For example, the accumulating sensations of pain and discomfort during a prolonged, high-intensity endurance task can encourage an individual to gradually focus on relieving the pain, and eventually the cons (i.e., pain) outweigh the pros (i.e., optimal performance) of continuing the endurance task and participants choose to quit (Taylor et al., 2018).

Support for these models comes from a substantial evidence base suggesting that performance on subsequent physical tasks is reduced following self-control exertion (e.g., Dorris et al., 2012; Wagstaff, 2014; Englert and Wolff, 2015). Typically, experimental protocols have consisted of two unrelated tasks requiring self-control, commonly referred to as the sequential-task paradigm (Baumeister et al., 2007). Within the sequential-task paradigm, the experimental (self-control) group/condition requires participants to exert self-control on both tasks. Conversely, in the control (non-self-control) group/condition, the initial task does not require any, or very little, self-control (Baumeister et al., 1998). Typically, the self-control tasks utilized require the alteration or modification of an instinctive, well-learned response, similar to resisting an impulse or temptation (Baumeister et al., 2007). Research suggests that when the initial task requires self-control, performance on the second self-control task will be impaired, relative to when the first task does not require self-control (Baumeister et al., 1998).

Within the sequential task paradigm, the duration of the initial self-control task appears inconsistent throughout the literature (Brown and Bray, 2017); however, the majority of the primary self-control tasks are relatively brief in duration (typically 4–15 min; Giboin and Wolff, 2019). In contrast, mental fatigue research utilizes initial tasks that are 30 min or longer, and typically ∼90 min in duration (e.g., Van Cutsem et al., 2017). Therefore, it has been argued that typical self-control depletion tasks are not long enough to lead to subjective feelings of mental fatigue (Pageaux et al., 2013). In addition, regarding self-control, all studies to date have only examined one duration of initial self-control exertion; research has not manipulated the initial task duration within the sequential-task paradigm, or considered the effect on physical performance during the second self-control task (Hagger et al., 2010; Lee et al., 2016; Giboin and Wolff, 2019). While recent research has demonstrated that different durations of the initial self-control task did not affect subsequent cognitive performance (Wolff et al., 2019), it is currently unknown whether longer durations of self-control exertion could have a greater detrimental effect on subsequent physical performance. Spending longer on the initial self-control task may lead to greater shifts in motivation and attention (Inzlicht and Schmeichel, 2016), exacerbating the performance decrements on a subsequent physical task, also requiring self-control.

Building on the literature discussed above, the aims of the current research were to explore: (a) the potential for the initial self-control task duration to moderate any decrements in performance on a subsequent physical task and (b) whether exerting self-control increases perceptions of pain and reduces perceptions of motivation during a subsequent physical task. Based on the broad self-control literature (e.g., Dorris et al., 2012; Inzlicht and Schmeichel, 2016; Boat and Taylor, 2017), it was hypothesized that spending longer on the initial self-control task would result in an increased deleterious effect on subsequent wall-sit task performance (hypothesis 1). In addition, it is hypothesized that self-control exertion will lead to increased perceptions of pain, and reduced perceptions of motivation, during the wall-sit task (hypothesis 2).

## Materials and Methods

### Participants

The sample consisted of 29 participants (11 male, 18 female) aged 18–22 years old (M age = 20.7 years, SD = 0.8 years). On average, the participants exercised on 3 days (SD = 2 days) per week. All participants were healthy, as determined by a University approved general health questionnaire. A power calculation (G^∗^Power version 3.1; Faul et al., 2007) with power = 0.95 and α = 0.05 (ANOVA repeated measures, within factors), specified a minimum sample size of *N* = 23 would be satisfactory to detect a medium effect size (0.40), which is representative of previous self-control studies (Giboin and Wolff, 2019; Brown et al., 2020).

### Procedures

Following ethical approval, the study was explained in full to participants (including that their participation was anonymous and voluntary). Participants then signed an informed consent form. In addition, participants were asked to refrain from strenuous physical activity and alcohol consumption for 24 h before the start of each trial. Participants took part in four experimental sessions in total (separated by at least 48 h).

#### Experimental Protocol

On arrival in the laboratory, participants first completed questionnaires to control for the influence of daily stress (see section “Measures”), given the potential for stress to influence the effects of self-control exertion on subsequent performance (Tangney et al., 2004; Englert and Rummel, 2016). Participants were then familiarized with the wall-sit procedure. Individuals were instructed to lean with their back against a wall, hips and knees bent at 90°, feet shoulder width apart, with their hands resting against the wall (Boat et al., 2018). This task requires self-control as the procedure becomes increasingly painful and requires individuals to persist at the task, rather than quit the wall-sit, to relieve the associated pain (Boat and Taylor, 2017; Boat et al., 2018). The physical task instructions were scripted so that they remained the same for all trials. Individuals practiced the wall-sit task once to ensure that they were familiar with it and understood the task requirements. This procedure has been used successfully in similar self-control research (e.g., Boat and Taylor, 2017; Boat et al., 2018).

Participants were then required to complete either a non-self-control task (congruent Stroop task) for 4 min, or a self-control task (incongruent Stroop task) for 4 (short duration), 8 (medium duration), or 16 min (long duration). Self-control manipulation took place via a modified Stroop task (Stroop, 1935), which is well established and commonly used in the self-control literature (e.g., McEwan et al., 2013; Englert and Wolff, 2015; Boat et al., 2017). Furthermore, these durations of the Stroop task were utilized as previous research has employed this task for the same length of time (i.e., 4 min; Boat and Taylor, 2017). Also, 8 and 16 min reflect a 200 and 400% increase in duration, respectively, thus reflecting a suitable variance for differences to be observed and is in line with previous research (e.g., Wolff et al., 2019).

In the Stroop task, a word (always a color) was displayed in the center of a computer screen, and participants were required to select the correct response using a response pad. In the congruent version of the Stroop task (non-self-control exertion), the word and the print color were congruent (e.g., the word “green” was printed in green ink). In the incongruent version of the Stroop task (self-control exertion), the word itself and the print color were incongruent. For instance, if the word “green” was printed in blue ink, the correct keypad response would be the blue button. The incongruent Stroop task requires self-control because participants have to inhibit their natural response to name the word rather than the ink color (e.g., McEwan et al., 2013; Englert and Wolff, 2015; Boat et al., 2018). Stimuli were presented on the screen one at a time, and remained until a response was registered. The Stroop task was completed in a quiet room and participants were asked to respond as quickly and as accurately as possible. Prior to the actual test, participants completed a brief (30 s) practice session to re-familiarize themselves with the requirements of the Stroop task. Immediately following the Stroop task, participants completed a manipulation check (CR-10 Scale; Borg, 1998), which assessed their perceived mental effort during the cognitive task (see section “Measures”).

Immediately following the completion of the CR-10 scale, participants performed the wall-sit. Participants were instructed to hold the position for as long as possible, until volitional exhaustion (i.e., the point at which participants chose to give up on the task, as they could no longer hold the correct wall-sit positioning). The time started as soon as participants were in the correct wall-sit position. The time was stopped when participant’s knees, extended above or flexed below, the required 90° angle they were asked to hold throughout the wall-sit. Overall, participants performed four wall-sits under four experimental conditions: non-self-control task (congruent Stroop task) for 4 min, or a self-control task (incongruent Stroop task) for 4 (short duration), 8 (medium duration), or 16 min (long duration). The order of the sessions was counterbalanced to eliminate order effects. Throughout the wall-sit task, participants’ perceptions of pain and motivation were recorded every 30 s (see section “Measures”).

### Measures

#### Daily Stress

The Daily Inventory of Stressful Events Questionnaire (Almeida et al., 2002) was utilized to measure participants’ daily stress. Participants were instructed to indicate whether or not a number of stressful events had occurred on the day (e.g., “Anything at work or university that most people would consider stressful”). This questionnaire has been shown to have high internal consistency and predictive validity (Almeida et al., 2002).

#### Mental Exertion

Borg’s single-item CR-10 scale (Borg, 1998) was completed to measure mental exertion following the Stroop task (0 = extremely weak; 10 = absolute maximum). This questionnaire has been used extensively in previous self-control research (e.g., McEwan et al., 2013; Boat et al., 2018).

#### Perceptions of Pain and Motivation

A Visual Analog Scale (VAS), adapted from the short-form McGill pain questionnaire (Melzack, 1987), was used to measure participant’s perceptions of pain, and motivation to continue the wall-sit task, every 30 s during the wall-sit. Both VAS scales consisted of a 10 cm line (“no pain” to “worst possible pain”; “zero motivation to continue” to “full motivation to continue”) with participants’ responding according to their perceived pain and motivation at that point in time. The VAS has demonstrated acceptable predictive validity and reliability (Wright et al., 2001) and has been successfully utilized in previous self-control research (e.g., Boat and Taylor, 2017; Boat et al., 2018).

#### Task Performance

Performance was measured using the time (in seconds) participants quit the wall-sit task. Quitting the wall-sit task was considered as the moment when participant’s knees, extended above or flexed below, the required 90° angle they were asked to hold the wall-sit.

### Statistical Analysis

Data were analyzed using SPSS (version 25; SPSS Inc., Chicago, IL, United States). To check for baseline differences between the trials, stress, fatigue, and mental exertion were analyzed using one-way repeated measures analysis of variance (ANOVA), with Bonferroni-corrected paired samples *t*-tests used as *post hoc* testing where significant differences existed. Wall-sit performance time was also analyzed using one-way repeated measures ANOVA (with Bonferroni-corrected paired samples *t*-tests as *post hoc* testing, with effect sizes calculated as Cohen’s *d*).

Due to the different number of data points between participants and experimental trials for perceptions of pain and motivation (given these were measured every 30 s), multi-level modeling was used to analyze these data. These analyses were conducted in the open-source software R (version 3.5.1^1^). First, data were transformed to ensure a normal distribution (due to the left-hand skew and right-hand skew of pain and motivation data, respectively). All parameter estimates were “untransformed” prior to reporting, for ease of interpretation. Subsequently, linear mixed effect models were applied using the *lme* function (which yields “*t*” statistics), utilizing a trial ^∗^ time approach, with a random effect (intercept) for each participant included in all models. To gain a greater insight, trial was converted to a factor, to allow comparisons between each of the experimental trials. Further separate linear mixed effect models were conducted for initial (i.e., at 30 s into the wall-sit task) perceptions of pain and motivation, due to the aforementioned evidence suggesting that shifts in pain and motivation may occur early in the wall-sit task (Boat and Taylor, 2017; Boat et al., 2018). Furthermore, to examine how initial pain and initial motivation affected wall-sit performance time, linear mixed effect models were conducted. For these models, the dependent variable was wall-sit performance time and the independent variables were trial, initial pain, and initial motivation. To compare model fit, Akaike information criteria (AIC) and Bayesian information criteria (BIC) were used, with smaller AIC and BIC values indicating that the independent variables explain a greater amount of the variance in the dependent variable. For all analyses, statistical significance was accepted as *p* < 0.05.

## Results

### Pre-trial Manipulation Checks

There was no difference at baseline between the trials for stress (*p* = 0.734) or fatigue (*p* = 0.388). However, the manipulation of self-control did affect mental exertion [main effect of trial, *F*_(3,84)_ = 77.1, *p* < 0.001]. Upon further inspection, pairwise comparisons revealed mental exertion was significantly different between all trials (non-self-control exertion: 0.8 ± 0.1; short duration self-control exertion: 2.5 ± 0.2; medium duration self-control exertion: 3.9 ± 0.3; long duration self-control exertion: 5.5 ± 0.4; all pairwise comparisons, *p* < 0.001). These findings confirm the manipulation of self-control.

### Wall-Sit Performance Time

**FIGURE 1:**
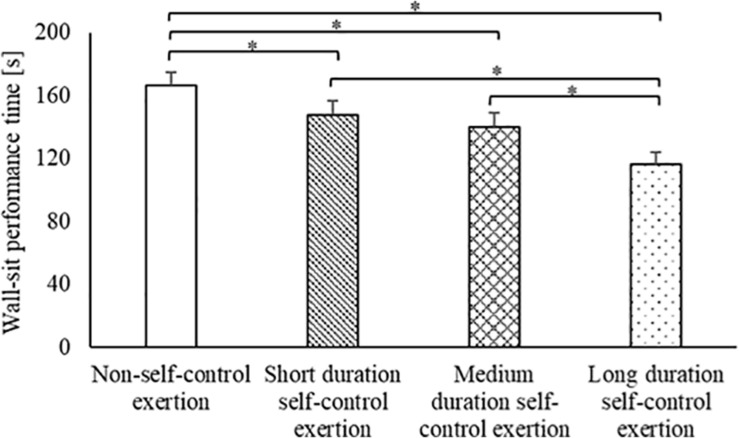
Wall-sit performance time on all trials. Data are mean ± SEM (main effect of trial, *p* < 0.001; * indicates difference between trials, *p* < 0.01).

Overall, wall-sit performance time was significantly different between the trials [main effect of trial, *F*_(3,84)_ = 22.7, *p* < 0.001; Figure 1]. Upon further inspection, wall-sit performance time was significantly longer on the non-self-control exertion trial (166 ± 9 s, range 98–305 s), compared to all other trials [short duration self-control exertion: 148 ± 9 s, range 74–263 s, *t*_(28)_ = 2.8, *p* = 0.008, *d* = 0.38; medium duration self-control exertion: 140 ± 9 s, range 71–295 s, *t*_(28)_ = 3.9, *p* = 0.001, *d* = 0.53; long duration self-control exertion: 116 ± 8 s, range 70–234 s, *t*_(28)_ = 9.4, *p* < 0.001, *d* = 1.13]. Wall-sit performance time was also significantly longer on both the short duration self-control exertion [*t*_(28)_ = 5.1, *p* < 0.001, *d* = 0.71] and medium duration self-control exertion [*t*_(28)_ = 4.6, *p* < 0.001, *d* = 0.53] trials, compared to the long duration self-control exertion trial. However, there was no difference in wall-sit performance time between the short duration and medium duration self-control exertion trials (*p* = 0.270, *d* = 0.16).

### Perceptions of Pain

Overall, there was a difference in perceptions of pain between the trials [main effect of trial, *t*_(474)_ = 3.2, *p* = 0.001; Table 1]. Upon further inspection, perceived pain was significantly greater on the medium duration self-control exertion [*t*_(474)_ = 2.2, *p* = 0.031] and long duration self-control exertion [*t*_(470)_ = 2.6, *p* = 0.011] trials, compared to the non-self-control exertion trial. There was no overall difference in perceived pain between the other trials (all *p* > 0.05). All models demonstrated that perceived pain increased across time on all trials (main effect of time, all *p* < 0.001). However, the pattern of change in perceived pain across time was similar between all trials (trial ^∗^ time interactions, all *p* > 0.05; Table 1).

**TABLE 1:** Results of the multilevel models conducted for perceptions of pain.

	Baseline trial	Comparison trial	Intercept	Parameter estimate	95% CI	*t*	*p*
Main effect of trial	Non-self-control exertion	Short duration self-control exertion	1.76	5.68	4.79, 6.53	3.17	0.002
		Medium duration self-control exertion		5.98	5.10, 6.81	16.40	< 0.001
		Long duration self-control exertion		6.25	5.30, 7.12	0.89	0.376
	Short duration self-control exertion	Medium duration self-control exertion	2.35	5.31	4.39, 6.21	0.66	0.507
		Long duration self-control exertion		5.59	4.59, 6.55	1.16	0.246
	Medium duration self-control exertion	Long duration self-control exertion	2.58	5.28	4.39, 6.16	0.56	0.579
Trial * time interaction	Non-self-control exertion	Short duration self-control exertion	1.76	5.02	4.77, 5.27	0.14	0.892
		Medium duration self-control exertion		4.97	4.71, 5.22	–0.25	0.802
		Long duration self-control exertion		5.29	4.96, 5.61	1.75	0.082
	Short duration self-control exertion	Medium duration self-control exertion	2.35	4.95	4.68, 5.22	–0.36	0.717
		Long duration self-control exertion		5.27	4.94, 5.60	1.58	0.115
	Medium duration self-control exertion	Long duration self-control exertion	2.58	5.32	4.98, 5.65	1.85	0.065
Initial pain	Non-self-control exertion	Short duration self-control exertion	3.59	0.40	−0.35, 1.15	1.04	0.303
		Medium duration self-control exertion		0.77	0.02, 1.52	2.00	0.049
		Long duration self-control exertion		1.19	0.44, 1.94	3.10	0.003
	Short duration self-control exertion	Medium duration self-control exertion	3.99	0.37	−0.38, 1.12	0.96	0.338
		Long duration self-control exertion		0.79	0.04, 1.54	2.06	0.042
	Medium duration self-control exertion	Long duration self-control exertion	4.36	0.42	-0.33, 1.17	1.10	0.275

#### Initial Perceptions of Pain

**FIGURE 2:**
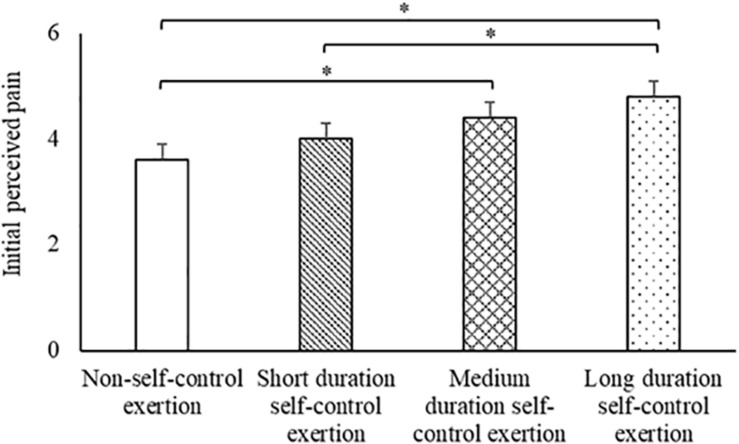
Initial (30 s) perceptions of pain across the trials. Data are mean ± SEM (main effect of trial, *p* = 0.001; * indicates difference between trials, *p* < 0.05).

When considering initial (30 s) perceived pain, there was a significant difference between the trials [main effect of trial, *t*_(86)_ = 3.3, *p* = 0.001; Figure 2]. Specifically, perceived pain was greater on the long duration self-control exertion trial (4.8 ± 0.3) compared to the non-self-control exertion trial [3.6 ± 0.3; *t*_(84)_ = 3.1, *p* = 0.003] and short duration self-control exertion trial [4.0 ± 0.3; *t*_(84)_ = 2.1, *p* = 0.042]; and was also greater on the medium duration self-control exertion trial (4.4 ± 0.3) compared to the non-self-control exertion trial [*t*_(84)_ = 2.0, *p* = 0.049]. All other pairwise comparisons for initial perceptions of pain revealed no differences between the trials (all *p* > 0.05).

### Motivation

Overall, there was a difference in motivation between the trials [main effect of trial, *t*_(474)_ = −2.8, *p* = 0.005; Table 2]. Upon further inspection, motivation was significantly greater on the non-self-control exertion trial compared to all other trials [main effects of trial: short duration self-control exertion, *t*_(470)_ = −2.7, *p* = 0.007; medium duration self-control exertion, *t*_(470)_ = −2.1, *p* = 0.037; long duration self-control exertion, *t*_(470)_ = −2.7, *p* = 0.008]. There was no overall difference in motivation between the self-control exertion trials (all *p* > 0.05). All models demonstrated that motivation decreased across time on all trials (main effect of time, all *p* < 0.001). The decrease in motivation across the wall-sit was greater on the long duration self-control exertion trial, compared to all other trials (trial ^∗^ time interactions: non-self-control exertion, *t*_(470)_ = −2.3, *p* = 0.022; short duration self-control exertion, *t*_(470)_ = −2.3, *p* = 0.023; medium duration self-control exertion, *t*_(470)_ = −2.1, *p* = 0.039; Table 2). The pattern of change in motivation across time was similar between the other trials (trial ^∗^ time interactions, all *p* > 0.05; Table 2).

**TABLE 2:** Results of the multilevel models conducted for motivation.

	Baseline trial	Comparison trial	Intercept	Parameter estimate	95% CI	*t*	*p*
Main effect of trial	Non-self-control exertion	Short duration self-control exertion	7.52	–3.43	−2.47, −4.54	–2.73	0.007
		Medium duration self-control exertion		–3.78	−2.76, −4.92	–2.09	0.037
		Long duration self-control exertion		–3.34	−2.32, −4.56	–2.65	0.008
	Short duration self-control exertion	Medium duration self-control exertion	6.13	5.38	4.18, 6.53	0.62	0.537
		Long duration self-control exertion		–4.90	−3.63, −6.19	–0.15	0.884
	Medium duration self-control exertion	Long duration self-control exertion	6.48	–4.53	−3.29, −5.83	–0.71	0.476
Trial * time interaction	Non-self-control exertion	Short duration self-control exertion	7.52	5.02	4.69, 5.34	0.11	0.916
		Medium duration self-control exertion		–4.98	−4.64, −5.31	–0.15	0.885
		Long duration self-control exertion		–4.51	−4.10, −4.93	–2.29	0.022
	Short duration self-control exertion	Medium duration self-control exertion	6.13	–4.96	−4.61, −5.31	–0.24	0.815
		Long duration self-control exertion		–4.94	−4.51, −5.38	–2.28	0.023
	Medium duration self-control exertion	Long duration self-control exertion	6.48	–4.54	−4.10, −4.98	–2.08	0.039
Initial pain	Non-self-control exertion	Short duration self-control exertion	6.49	–1.53	−0.63, −2.44	–3.32	0.001
		Medium duration self-control exertion		–1.52	−0.62, −2.43	–3.29	0.001
		Long duration self-control exertion		–2.33	−1.43, −3.24	–5.04	<0.001
	Short duration self-control exertion	Medium duration self-control exertion	4.95	0.01	−0.90, 0.92	0.02	0.982
		Long duration self-control exertion		–0.80	−1.71, 0.11	–1.73	0.088
	Medium duration self-control exertion	Long duration self-control exertion	4.96	–0.81	−1.72, 0.10	–1.75	0.084

#### Initial Perceptions of Motivation

**FIGURE 3:**
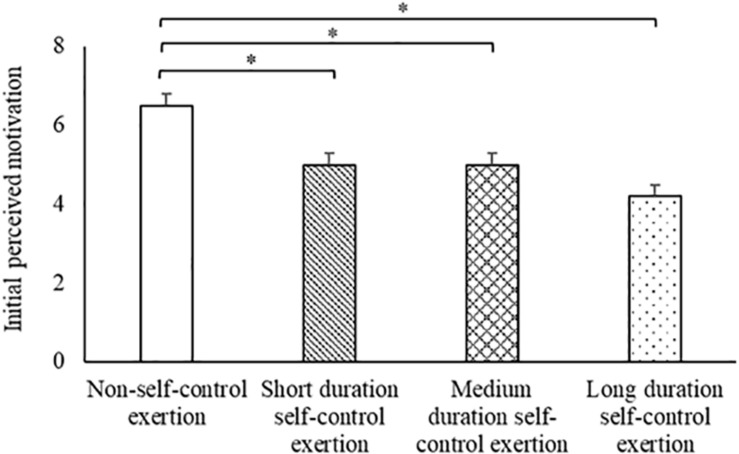
Initial (30 s) perceptions of motivation across the trials. Data are mean ± SEM (main effect of trial, *p* < 0.001; * indicates difference between trials, *p* < 0.01).

When considering initial (30 s) motivation, there was a significant difference between the trials [main effect of trial, *t*_(86)_ = −4.7, *p* < 0.001; Figure 3]. Specifically, motivation was greater on the non-self-control exertion trial (6.5 ± 0.3) compared to all other trials [main effect of trial: short duration self-control exertion, 5.0 ± 0.3, *t*_(84)_ = −3.3, *p* = 0.001; medium duration self-control exertion, 5.0 ± 0.4, *t*_(84)_ = −3.3, *p* = 0.001; long duration self-control exertion, 4.2 ± 0.4, *t*_(84)_ = −5.0, *p* < 0.001]. All other pairwise comparisons for initial motivation revealed no differences between the trials (all *p* > 0.05).

### Factors Affecting Wall-Sit Performance Time

Table 3 presents the models examining how initial pain and initial motivation affected wall-sit performance time. The addition of initial pain and initial motivation separately to models 2 (AIC = 1113.6; BIC = 1127.2) and 3 (AIC = 1131.7; BIC = 1145.3), respectively, reduced the AIC and BIC compared to model 1 (AIC = 1134.4; BIC = 1145.4), indicating that both variables explain some of the variance in wall-sit performance time. Furthermore, the addition of initial pain and initial motivation to the same model (model 4) reduced the AIC and BIC further (AIC = 1111.6; BIC = 1127.9), suggesting that both of these variables contribute to explaining the variance in wall-sit performance time.

**TABLE 3:** Model characteristics examining the factors affecting wall-sit performance time.

Model	Variable	*p*	AIC	BIC
1: Trial	Trial	< 0.001	1134.4	1145.4
2: Trial + initial pain	Trial	< 0.001	1113.6	1127.2
	Initial pain	< 0.001		
3: Trial + initial motivation	Trial	< 0.001	1131.7	1145.3
	Initial motivation	0.139		
4: Trial + initial pain + initial motivation	Trial	< 0.001	1111.6	1127.9
	Initial pain	< 0.001		
	Initial motivation	0.256		

*AIC = Akaike information criteria; BIC = Bayesian information criteria.*

## Discussion

The present study examined the potential for the initial self-control task duration to moderate any decrements in performance on a subsequent physical task, and whether exerting self-control increased perceptions of pain and reduced motivation during a subsequent physical task. The findings provide novel evidence that spending longer on the initial self-control task led to greater detrimental effects on subsequent wall-sit performance time. Furthermore, a longer duration self-control exertion task led to increased perceptions of pain and decreased motivation within the first 30 s of, as well as a greater decrease in motivation across, the wall-sit task. Perceptions of pain and motivation may explain decrements in physical performance following the exertion of self-control.

A key finding of the present study was that a relatively brief (4 min) self-control exertion task led to impaired performance on a subsequent physical (wall-sit) task. Participants gave up quicker following a difficult cognitive task (requiring self-control), compared to when they completed a cognitively simple task (requiring no self-control). This is supported by previous research also demonstrating that a relatively brief self-control exertion task (i.e., 4–6 min) affects subsequent physical performance (e.g., Englert and Wolff, 2015; Boat and Taylor, 2017; Brown and Bray, 2017; Boat et al., 2018). Moreover, the findings significantly extend the extant literature by providing novel evidence that spending longer on the initial self-control task led to greater detrimental effects on subsequent wall-sit performance time. Participants persisted at the wall-sit task 32 s longer on average, when they exerted self-control for a short duration (i.e., 4 min) relative to when they exerted self-control for a long duration (i.e., 16 min); equivalent to a 28% improvement in performance. This is interesting given that recent research has suggested that the initial task duration is not associated with the magnitude of performance impairment for physical (Giboin and Wolff, 2019) or cognitive (Wolff et al., 2019) performance. However, it is important to highlight that prior cognitive exertion appears to have a greater negative influence on performance during subsequent isolation tasks (e.g., wall-sit task), compared to whole-body endurance tasks (e.g., cycling) (Giboin and Wolff, 2019). As such, varying physiological and psychological task demands may well contribute to this debate. Future studies could also examine the effects on “real world” sporting performance by employing ecologically valid physical endurance tasks that require self-control (e.g., cycling). This study provides initial evidence that longer durations of self-control exertion have a greater negative impact on subsequent physical performance. It is possible that differences in the size of the depletion effect across previous studies may well be a result of the variations in the duration of the initial self-control task (Lee et al., 2016).

Another key finding of the present study was that the exertion of self-control led to elevated perceptions of pain and reduced motivation during the first 30 s of the wall-sit task. These findings are in accordance with previous research (e.g., Boat and Taylor, 2017; Boat et al., 2018) and align well with the shifting priorities model of self-control (Inzlicht et al., 2014; Inzlicht and Schmeichel, 2016), whereby self-control exertion led to a state of elevated distress in the early stages of the wall-sit task (Elkins-Brown et al., 2017). This aversive state has been proposed to not only encourage individuals to attend to the presence of task goal conflict (i.e., quitting to relieve the pain versus persisting on the wall-sit task) (Baumeister and Bargh, 2014), but also encourage participants to prepare for actions to reduce this distressing state (Inzlicht and Legault, 2014). Accordingly, motivational priorities shifted toward an increased focus on the proximal tempting goal (i.e., quitting or reducing effort on the wall-sit task to relieve the pain), relative to the distal goal (i.e., persisting on the wall-sit task to optimize performance), resulting in reductions in performance following self-control exertion, in line with the shifting priorities (Inzlicht and Schmeichel, 2016; Milyavskaya and Inzlicht, 2018) and reward-based (Kurzban et al., 2013; Wolff and Martarelli, 2020) models of self-control. Of note, the findings of the present study suggest that both initial pain and initial motivation contribute to explaining the variance in wall-sit performance time following the depletion of self-control.

Previous research has only examined the effects of self-control exertion on perceptions of pain and motivation at the very early and final stages of the subsequent physical performance task (e.g., Boat and Taylor, 2017; Boat et al., 2018). The present study extends these findings by examining perceptions of pain and motivation throughout the wall-sit task, with the findings suggesting that participant’s motivation decreased more rapidly during the wall-sit task on the long duration self-control exertion trial (i.e., 16 min). However, there were no differences in the pattern of change in perceptions of pain throughout the wall-sit task across the experimental trials. These findings imply that perceptions of pain and motivation in the early stages of the wall-sit task are a potential mechanism to explain the performance decrements following prior self-control exertion. The findings of the present study also suggest that long durations of self-control exertion influence motivation throughout the subsequent physical performance task. This novel finding has implications for the design of future interventions aimed at attenuating the effects of self-control exertion on subsequent physical performance. Intervention strategies that target motivation throughout subsequent physical tasks, by reinforcing the value of distal goals (e.g., persisting on a physical task to optimize performance), or decreasing the worth of indulging in competing proximal goals (e.g., quitting or reducing effort on the physical task to relieve the pain) may help to reduce the rapid decline in motivation following self-control exertion (Taylor et al., 2018). Specifically, the findings of the present study suggest that future interventions should target initial perceptions of pain and motivation, as well as motivation throughout the subsequent physical task, to target the tenants of the shifting priorities model that were affected in the present study and ultimately enhance physical performance.

### Limitations and Future Research Directions

Although yielding important findings, some limitations must be addressed. For example, performance on the initial self-control task (i.e., the Stroop task) was not examined. It is possible that individuals may have exerted differing amounts of self-control according to the extent to which they were motivated during the initial self-control task (Lee et al., 2016). While the CR-10 questionnaire confirmed the manipulation of self-control in the present study, monitoring performance on the Stroop task could provide an informative measure of participants’ engagement and motivation during the initial self-control task (Lee et al., 2016). However, recent evidence has indicated that performance does not vary across different durations of the Stroop task (Wolff et al., 2019). In addition, although the participants in the current study were recreationally active (three times per week), we did not assess details of participants habitual physical activities. Future research could explore how habitual exercise habits may mediate the effects of self-control depletion on subsequent physical performance.

It is important to highlight that in the current study we utilized a 4-min control task (i.e., congruent Stroop task) as the reference performance for all self-control depleting conditions. Future research could compare self-control depleting tasks with the same duration (i.e., 8-min congruent Stroop task vs. 8-min incongruent Stroop task) to provide further insight into the potential for the duration of the initial self-control task to influence subsequent physical performance, perceptions of pain, and perceived motivation.

Furthermore, our findings are in line with the tenants of the shifting priorities model of self-control from a motivational and attentional viewpoint (Inzlicht et al., 2014; Inzlicht and Schmeichel, 2016). However, the use of objective measures of perceived pain and motivation may yield valuable insights into these underpinning mechanisms of the shifting priorities model. For example, electroencephalogram (EEG) and fNIRS activity of the prefrontal cortex could be utilized to examine the underlying motivational processes (Schmeichel et al., 2016). In addition, electromyography (EMG) of the facial muscles could be used to objectively measure perceptions of effort and pain (Huang et al., 2014), as well as eye-tracking to explore attentional focus (Kredel et al., 2017). Consequently, such methods would enable the objective exploration of shifts in motivational and attentional processes, following self-control exertion, while completing physically demanding tasks.

Finally, researchers should investigate additional mechanisms that may explain performance reductions following self-control exertion. For instance, recent research has suggested that within the sequential task paradigm, the initial self-control task is likely to induce forms of boredom, thus altering behavior and influencing performance on subsequent tasks that require self-control (Milyavskaya et al., 2019; Wolff and Martarelli, 2020). As such, task-induced boredom could be further investigated as a psychological factor that may explain performance reductions following self-control exertion.

## Conclusion

The present study provides novel evidence that spending longer on the initial self-control task leads to greater detrimental effects on subsequent wall-sit performance time. Furthermore, the present study suggests that a longer duration self-control exertion task leads to increased perceptions of pain and decreased perceptions of motivation within the first 30 s of the wall-sit task, as well as a greater decrease in motivation across the wall-sit task. These attentional and motivational shifts may explain performance decrements following the exertion of self-control.

## Data Availability Statement

The raw data supporting the conclusions of this article will be made available by the authors, without undue reservation.

## Ethics Statement

The studies involving human participants were reviewed and approved by the Nottingham Trent University Ethics Committee. The patients/participants provided their written informed consent to participate in this study.

## Author Contributions

RB and SC designed the study and analyzed and interpreted the data. RB, RH, EW, AD, and ET collected the data. RB, RH, and SC drafted and revised the manuscript. All authors approved the final version of the manuscript.

## Conflict of Interest

The authors declare that the research was conducted in the absence of any commercial or financial relationships that could be construed as a potential conflict of interest.

## Footnotes

1 www.r-project.org
